# A Multicenter, Retrospective Comparison Study of Pregnancy Outcomes According to Placental Location in Placenta Previa

**DOI:** 10.3390/jcm13030675

**Published:** 2024-01-24

**Authors:** Seon Ui Lee, Ji Hye Jo, Haein Lee, Yoojin Na, In Yang Park

**Affiliations:** 1 Department of Obstetrics and Gynecology, St. Vincent’s Hospital, College of Medicine, The Catholic University of Korea, Seoul 16247, Republic of Korea; 2 Department of Obstetrics and Gynecology, College of Medicine, The Catholic University of Korea, Seoul 06591, Republic of Korea; 3 Department of Obstetrics and Gynecology, Seoul St. Mary’s Hospital, College of Medicine, The Catholic University of Korea, Seoul 06591, Republic of Korea; 4 Department of Obstetrics and Gynecology, Yeouido St. Mary’s Hospital, College of Medicine, The Catholic University of Korea, Seoul 07345, Republic of Korea

**Keywords:** abnormal placentation, obstetric outcome, placenta accreta spectrum, ultrasonography

## Abstract

**Background:** We investigated the association between placental location and pregnancy outcomes in placenta previa. **Methods:** This multi-center retrospective study enrolled 781 women who delivered between May 1999 and February 2020. We divided the dataset into anterior (*n* = 209) and posterior (*n* = 572) groups and compared the baseline characteristics and obstetric and neonatal outcomes. The adverse obstetric outcomes associated with placenta location were evaluated using a multivariate logistic analysis. **Results:** Gestational age at delivery in the anterior group (253.0 ± 21.6) was significantly lower than that in the posterior group (257.6 ± 19.1) (*p* = 0.008). The anterior group showed significantly higher parity, rates of previous cesarean section, non-vertex fetal positions, admissions for bleeding, emergency cesarean sections, transfusions, estimated blood loss, and combined placenta accrete spectrum (*p* < 0.05). In the multivariate analysis, the anterior group had higher rates of transfusion (OR 2.23; 95% CI 1.50–3.30), placenta accreta spectrum (OR 2.16; 95% CI 1.21–3.97), and non-vertex fetal positions (OR 2.47; 95% CI 1.09–5.88). **Conclusions:** These findings suggest that more caution is required in the treatment of patients with anterior placenta previa. Therefore, if placenta previa is diagnosed prenatally, it is important to determine the location of the body and prepare for massive bleeding in the anterior group.

## 1. Introduction

Placenta previa (PP) is an obstetric complication in which the placenta completely or partially overlies the endocervical os [1]. Historically, PP was first diagnosed when a pregnant patient had painless vaginal bleeding. However, this condition can now be detected through routine ultrasound examinations during pregnancy [2]. Most cases of PP diagnosed by second-trimester ultrasound resolve by the third trimester, but 10 to 20% of cases persist until delivery [2]. The global prevalence of PP has been estimated to be 5.2 per 1000 pregnancies of all term gestations [1].

Although its pathophysiology remains unclear, its occurrence is significantly associated with uterine scarring and endometrial damage [3]. The risk factors for PP include the use of uterine instrumentation (e.g., curettage), previous PP, and a previous cesarean section [4,5]. In particular, with an increasing number of previous cesarian deliveries, a dose–response pattern has been observed in the risk of PP [1]. Other risk factors include advanced maternal age, multiparity, chronic hypertension, diabetes, smoking, cocaine use during pregnancy, multiple gestations, and the use of assisted reproductive technology (ART) [4,5,6]. The incidence of PP continues to rise with the increasing rates of cesarean delivery and ART.

Women with PP have an approximately 10-fold higher risk of antenatal vaginal bleeding. The mechanism underlying bleeding remains unknown but appears to be attributed to the separation of the placenta from the underlying decidua resulting from contraction, cervical effacement, cervical dilatation, and advancing gestational age [1].

Since PP usually manifests as antepartum bleeding, neonatal morbidity and mortality are also common and result primarily from premature birth. Neonatal complications are primarily associated with premature infants. According to a population-based study, 55.6% of women with placenta previa delivered after 37 weeks of gestation, 27.5% delivered between 34 and 37 weeks of gestation, and 16.9% delivered before 34 weeks of gestation [7]. As a result, perinatal mortality increased by three or four fold [8].

Pregnant women with PP usually undergo a cesarean section, and PP is a major cause of intrapartum and postpartum hemorrhage [6,9]. The major complications encountered include hemorrhage, requirement for blood transfusion, intensive care admissions, uterine artery embolization (UAE), hysterectomy, and even maternal death.

Moreover, PP is also major risk factor for placenta accreta spectrum (PAS), which is a spectrum of diseases that intensify the severity of postpartum hemorrhage [10]. PAS is a group of diseases in which placental tissue invades the myometrium. In 2019, the International Federation of Gynecology and Obstetrics (FIGO) published a guideline for grading the depth of invasion based on intraoperative and pathological diagnoses [11].

Because heavy vaginal bleeding is quite possible, the American College of Obstetricians and Gynecologists (ACOG) advocates for scheduled cesarean delivery of pregnant women diagnosed with PP at 36 0/7–37 6/7 gestational weeks; therefore, the neonatal risks of late-preterm and early-term births are acknowledged as an acceptable risk to avoid emergent delivery because of bleeding [12].

The placenta transports all nutrients, oxygen, and fluids from the mother to the fetus and removes fetal waste [13]. It also provides important information about the cause and timing of many adverse outcomes including neurological damage, fetal distress, infection, intrauterine growth restriction (IUGR), and death and aids in the identification of unexpected maternal disorders and primary placental disorders [14].

If the placenta is widely located including along the anterior wall of the uterus it is difficult to avoid transecting the placenta. Direct incision of the placenta triggers the need for rapid delivery and occurrence of sudden, extensive maternal blood loss [3].

Although there has been extensive research into abnormal placentation (placenta accreta) and low placental implantation, only a few studies have evaluated the other aspects of placental position and the impact they may have on pregnancy and neonatal outcomes [15,16,17,18,19]. Therefore, the aim of this study was to evaluate pregnancy outcomes according to placental location in women with PP.

## 2. Materials and Methods

### 2.1. Data Source and Ethical Considerations

A retrospective chart review was performed for all cases of PP. In addition, subsequent pregnancy-related records were extracted from the Clinical Data Warehouse of the Catholic Medical Center-affiliated hospital. The data extraction and utilization plan for this study were approved by the Central Institutional Review Board of the Catholic Medical Center (KC22RID0401). The study was conducted in accordance with the guidelines of the Declaration of Helsinki, and the rights of all the patients were protected.

### 2.2. Eligibility Criteria and Group Definition

**Figure 1:**
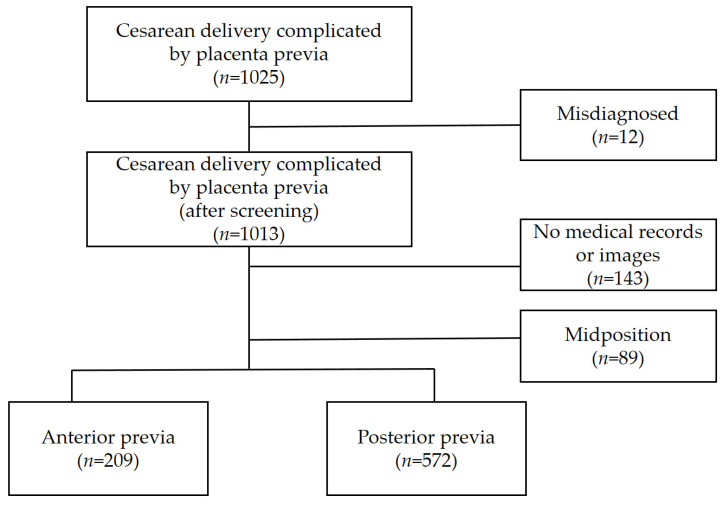
Flow chart depicting the selection and grouping study population.

This retrospective cohort study included cases wherein cesarean deliveries were performed because of PP at three university hospitals (Seoul St. Mary’s Hospital, Yeouido St. Mary’s Hospital, and St. Vincent’s Hospital in South Korea) between May 1999 and February 2020. The study included women who underwent cesarean section after a prenatal ultrasound diagnosis of PP (Figure 1). The patients were categorized into two groups based on the location of the placenta, as diagnosed using ultrasound: anterior and posterior groups. The anterior group was defined as women with more than 50% of placental tissue attached to the anterior wall. The posterior group was defined as women with more than 50% of placental tissue attached to the posterior wall.

The maternal demographic characteristics included maternal age, body mass index (BMI), parity, gestational age at delivery, history of intrauterine pregnancy (IUP), previous cesarean section, previous uterine surgery other than cesarean section, previous postpartum hemorrhage (PPH), and previous diagnosis of PP. The measured outcome parameters included indications for cesarean section, preoperative and postoperative hemoglobin (Hb) levels, emergent surgery, transfusion during and after surgery, estimated blood loss, accompanying placental problems (accreta or abruption), PPH, disseminated intravascular coagulation (DIC), and intensive care unit (ICU) admission. Neonatal birth weight, Apgar score, fetal sex, and fetal presentation were included as neonatal outcomes.

The possible risk factors for adverse outcomes in pregnant women with anterior PP were analyzed using a multivariate regression analysis. The evaluated factors were related to transfusion, massive transfusion (packed RBC > 3 or 5), placenta accreta, admission during pregnancy, and non-vertex fetal presentation.

Information on estimated blood loss was obtained from anesthesia records and the operative reports. The diagnosis of placenta accreta spectrum (PAS) was based on surgical and placental pathological findings. Postpartum hemorrhage was defined by the requirement for uterine artery embolization or intrauterine balloon insertion.

As this was a retrospective cohort study and because all data were anonymized, the need for informed consent was waived.

### 2.3. Statistical Analysis

This study aimed to determine whether there were any differences in pregnancy outcomes according to the location of the placenta. Therefore, confounding variables were identified by preferentially performing a homogeneity test to determine whether all baseline variables, other than the location of PP, were the same between the two groups. Variables for which homogeneity was not secured were separated by their level, and the resulting variables were compared. All continuous variables were tested for normality. When normality was secured, the means were compared using the Student’s *t*-test, and when normality was not secured, the Wilcoxon rank sum test or Mann–Whitney U test was performed. For categorical variables, the Chi-square test was used as a basis; however, when the number of samples in a specific category was insufficient, Fisher’s exact test was used to test the difference in the distribution of outcome variables between the two groups. Variables with significant differences in the results of univariate analyses were included in multivariable stepwise logistic regression models. The significance level for all statistical tests was set at *p* < 0.05. All analyses were performed using the R software (Version 4.1.2, R Foundation, Vienna, Austria).

## 3. Results

### 3.1. Study Population

During the study period, both delivery and neonatal records were obtained for 1013 cases. The location of the placenta was confirmed through a retrospective analysis of data through chart and image reviews. In 143 cases, it was difficult to confirm the location of the placenta using medical records and ultrasound images. After excluding missing data and records with a midposition placenta, 781 women were included in this study. The anterior group comprised 209 (26.8%) women and the posterior group comprised 572 (73.2%) women. The flow chart of the selection and grouping of the study population is presented in Figure 1.

### 3.2. Baseline Characteristics

Table 1 lists the baseline characteristics of the two groups. The anterior PP group showed significantly higher rates of previous cesarean sections, previous number of deliveries, abortion history, and IUP history (*p* < 0.05). Gestational age at delivery was significantly lower in the anterior PP group than that in the posterior group (253.0 ± 21.6 days (anterior group); 257.6 ± 19.1 (posterior group); *p* = 0.008). There was also a higher rate of admission bleeding in the anterior PP group (45.5% (anterior group) vs. 36.7% (posterior group); *p* = 0.027). The emergency cesarean section rate (due to bleeding, labor, and fetal distress) was significantly higher in the anterior PP group (*p* = 0.015). However, previa type, previous placenta previa, previous PPH, and previous uterine surgery except for cesarean section showed no significant differences between the two groups.

### 3.3. Pregnancy Outcomes

Table 2 shows the pregnancy outcomes of the two groups. No significant differences were observed between the preoperative and postoperative Hb levels between the two groups. The anterior group was more likely to receive a blood transfusion (57.4% (anterior group) vs. 36.7% (posterior group); *p* < 0.001). The transfusion rates for 5 or more and 10 or more units of red blood cells (RBCs) were significantly higher in the anterior group (5 or more, *p* = 0.002; 10 or more, *p* < 0.001). During the cesarean section, placental accreta spectrum (PAS) with PP was observed significantly more frequently in the anterior group (19.1% (anterior group) vs. 8.8% (posterior group); *p* < 0.001). Moreover, the EBL was higher in the anterior PP group (19.1% (anterior group); 8.8% (posterior group); *p* < 0.001). There were no statistically significant differences in terms of placental abruption, procedures for postpartum hemorrhage (including insertion of intrauterine balloon tamponade or uterine artery embolization), DIC, and ICU admission.

### 3.4. Neonatal Outcomes

Table 3 lists the neonatal outcomes in the two groups. As mentioned earlier, cesarean sections were performed at an earlier gestational age in the anterior group. The neonatal birth weight was significantly lower in the anterior group (2689.3 ± 704.9 g (anterior group) vs. 2815.7 ± 623.5 g (posterior group); *p* = 0.038). There were no significant differences in neonatal sex between the two groups. Both groups had slightly more male neonates. In addition, there were no significant differences in Apgar scores at 1 min and 5 min. In the anterior group, more cases were reported to have a non-vertex fetal presentation, such as a breech and transverse position (20.4% (anterior group) vs. 7.6 (posterior group); *p* < 0.001).

### 3.5. Multivariable Regression Analysis

A multivariable logistic regression analysis was performed to identify the risk of adverse outcomes with anterior PP, such as blood transfusions, massive transfusions (defined as transfusion of packed RBC > 3 or >5 units), placenta accreta, admissions to hospital during pregnancy, and non-vertex fetal presentations (Table 4). Anterior PP significantly increased the risk of transfusion (OR = 2.23; 95% CI: 1.50–3.33; *p* < 0.001), placenta accreta (OR = 2.16; 95% CI: 1.21–3.97; *p* = 0.009), and non-vertex fetal presentation (OR = 2.47; 95% CI: 1.09–5.88; *p* = 0.031).

## 4. Discussion

The objective of this study was to compare perinatal complications based on the location of the placenta in PP. This study has some important findings. First, high maternal parity and a history of cesarean section were associated with a greater occurrence of PP in the anterior part. Second, transfusions in anterior PP were more common than that in posterior PP. Third, anterior PP is more likely to accompany PAS.

The following are known risk factors for the development of PP: maternal age > 35 years, multiparity, smoking, prior cesarean delivery, multifetal gestation, and ART [12]. We found that the occurrence of anterior PP was higher in patients with a history of intrauterine pregnancy. This includes not only full-term or preterm delivery, but also miscarriages. It is well known that a previous cesarean section is a risk factor for the development of PP [20]. Lal et al. concluded that among women diagnosed with PP at second trimester screenings, PP was less likely to resolve in those who had a previous cesarean section. In their study, the resolution rate of PP at delivery for the prior cesarean section group was 61%, while for the no prior cesarean section group, it was 90% [21]. This study went one step further and found that a previous cesarean section was associated with anterior PP.

PP can cause hemorrhagic complications in the second half of pregnancy; therefore, caution is required during pregnancy. Many studies have associated bleeding and morbidity with PP. For example, PP or abruptio placentae have an adjusted odds ratio of 7.0 (95% CI: 6.6–7.3) for severe postpartum hemorrhage [22]. Karen et al. reported that PP was associated with an overall increased risk of maternal hemorrhagic morbidity (aRR 2.6, 95% CI: 1.9–3.5) [20].

We found that patients with anterior PP are more at risk of bleeding and requiring a transfusion. This study demonstrated that the incidence of transfusion is much higher in anterior PP than in posterior PP. There was also a significant difference between the two groups in the case of massive transfusions, such as five packs of red blood cells and ≥10 packs. Additionally, hospitalization due to bleeding during pregnancy was higher in the anterior group. Previous studies found that anterior PP was associated with a higher incidence of PPH, failure of progression, and later onset of labor. These findings support the hypothesis that an anterior placental location influences the mechanisms of uterine contractility [23].

Naturally, the risk of hemorrhage increases further if PP is complicated by placenta accreta or morbidly adherent placenta [24,25,26]. Unfortunately, the incidence of PAS in pregnant women with PP is higher than in those without PP [11]. A combination of PP and PAS can cause catastrophic bleeding and life-threatening conditions. Hong et al. suggested that when placentation occurs on the anterior wall and at a low position, it is similar to the covering of a cesarean section scar. The endometrium is damaged, and the muscular layer is weakened around the incision site. If villi are transplanted here, the underlying decidua may not form properly, allowing trophoblast cells to directly invade the myometrium. The villi would adhere or implant or penetrate the myometrium [27]. In a study that investigated and compared the degree and position of placentation, anterior placentation was found to be an independent risk factor for invasive PAS compared to posterior placentation [27]. This is similar to our findings, indicating a higher rate of transfusion with concomitant PAS. Recently, interest and research in PAS have increased globally. A previous study found that the placenta accreta index (PAI) had high clinical utility in predicting the risk of adherent placenta in PP. In the prediction model established in this study, two or more of cesarean sections, lacunae (more than grade 2), sagittal smallest myometrial thickness via ultrasonography, anterior PP, and bulging vessels to the bladder were used as significant predictors of placenta accreta [28].

This study is valuable as few studies have investigated the incidence of PAS according to the location of PP [27,29]. Based on the results of this study, surgeons need to be prepared for situations where they may encounter PAS when they have information about a patient with anterior PP.

It is already well known that placental magnetic resonance imaging (MRI) is another major tool for the antenatal diagnosis of PAS [30,31,32]. The sensitivity of MRI was 94.4% (95% CI, 86.0–97.9) and the specificity was 84.0% (95% CI, 76.0–89.8), which are similar to those of ultrasonography [33]. However, MRI studies are more prone to selection bias than ultrasonography studies are; thus, these data should be interpreted with caution. This is because, typically, only patients with inconclusive ultrasound findings or those at very high risk of placenta accreta spectrum undergo MRI [10]. MRI is more expansive and less widely used. Therefore, MRI is recommended only when the diagnosis is ambiguous after ultrasonography. Anterior PP has a relatively higher incidence of PAS than posterior PP, but visualization is relatively easy using ultrasonography. Therefore, the results of this study may be helpful in the clinical diagnosis of PAS. In this study, only six patients with PP underwent MRI (one of anterior group, five of posterior group) and four of them were diagnosed with PAS through MRI, which was consistent with the final pathological findings.

Obstetricians empirically know that more delicate techniques are required for anterior PP during cesarean section. Our study revealed that the occurrence of non-vertex fetal presentation (breech, transverse position, etc.) rather than vertex presentation was higher in anterior PP compared to that in posterior PP. This is also a factor that makes cesarean sections difficult for surgeons. In this study, the emergency cesarean section rate was higher in the anterior PP group. Furthermore, the anterior group delivered earlier than did the posterior group. The effect of low gestational age in anterior PP is thought to be due to the early intervention of antenatal complication (bleeding, fetal distress, and labor) and severe placental implantation.

Several guidelines provide recommendations regarding the timing of planned cesarean delivery in PP [34]. The Royal College of Obstetricians and Gynaecologists (RCOG) recommends that delivery timing should be tailored according to antenatal symptoms for PP [35]. Late preterm delivery (34 + 0 to 36 + 6 weeks of gestation) should be considered for women presenting with complicated PP, including a history of vaginal bleeding or other associated risk factors for preterm delivery. For women presenting with uncomplicated PP, delivery should be considered between 36 + 0 and 37 + 0 gestational weeks. The Society of Obstetricians and Gynecologists of Canada (SOGC) recommends cesarean delivery for PP at 36 + 0 to 36 + 6 weeks of gestation in the presence of risk factors and at 37 + 0 to 37 + 6 weeks of gestation in the absence of risk factors [36]. Because anterior placentation carries a higher risk of complications, cesarean section could be performed earlier than specified in the guidelines.

This study had some limitations, including its retrospective nature. Therefore, a lack of ability to control for potential confounding factors, such as underlying maternal disease and information bias, cannot be overlooked. To overcome this limitation, we performed a logistic regression analysis after adjusting for the variables. Despite these limitations, the study has several strengths. We obtained data from three centers in different regions. In addition, this study is meaningful as it focused on the main location of the placenta in PP. Although many studies have shown an association between PP and maternal hemorrhagic morbidity, studies focused on pregnancy outcomes according to the location of the placenta are rare [5,9]. Previous studies have focused on only major or minor types of PP [6]. This study can help improve pregnancy and postpartum outcomes. Ideally, a large, prospective randomized trial is needed to verify the clinical significance of the placental location in terms of pregnancy outcomes.

## 5. Conclusions

We found that anterior PP is an independent risk factor for PAS and the requirement for a transfusion. This suggests that more caution is needed in the treatment of patients with anterior PP. Therefore, if PP is diagnosed prenatally, it is important to determine the location of the body and prepare for massive bleeding in anterior PP. The results of this study appear to have the potential to improve pregnancy outcomes.

## Figures and Tables

**Table 1:** Baseline characteristics of the anterior and posterior groups.

Characteristic	Location of the Placenta		*p*-Value
	Anterior, *n* = 209	Posterior, *n* = 572	
Maternal age (years)	33.2 ± 4.2	33.5 ± 3.9	0.49
Abortion (%)	100 (47.8%)	228 (39.9%)	0.045
History of delivery ^1^ (%)	124 (59.3%)	275 (48.1%)	0.005
Parity (%)			<0.001
0	85 (40.7%)	297 (51.9%)	
1	87 (41.6%)	231 (40.4%)	
≥2	37 (17.7%)	44 (7.7%)	
IUP ^2^ (%)	157 (75.1%)	363 (63.5%)	0.002
Number of IUPs (%)			<0.001
0	52 (24.9%)	209 (36.5%)	
1	60 (28.7%)	182 (31.8%)	
≥2	97 (46.4%)	181 (31.6%)	
Gestational age at delivery (day)	253.0 ± 21.6	257.6 ± 19.1	0.008
Pre-pregnancy BMI (kg/m^2^)	21.3 ± 2.8	21.1 ± 2.9	0.43
CS Indication (%)			0.015
Bleeding	72 (34.4%)	150 (26.2%)	
Labor	20 (9.6%)	45 (7.9%)	
Fetal distress	11 (5.3%)	17 (3.0%)	
Elective	89 (42.6%)	322 (56.3%)	
Others	17 (8.1%)	38 (6.6%)	
Previous CS (%)	73 (34.9%)	114 (19.9%)	<0.001
Admission for bleeding (%)	95 (45.5%)	210 (36.7%)	0.027
Previa type (%)			0.218
Low lying	78 (37.3%)	188 (32.9%)	
Marginal	27 (12.9%)	111 (19.4%)	
Partialis	22 (10.5%)	66 (11.5%)	
Complete	81 (38.8%)	205 (35.8%)	
Vasa previa	1 (0.5%)	2 (0.3%)	
Previous placenta previa (%)	3 (1.4%)	6 (1.0%)	0.707
Previous uterine surgery except CS (%)	4 (1.9%)	12 (2.1%)	>0.999
Previous PPH (%)	1 (0.5%)	1 (0.2%)	0.464

The values are numbers (percentages) or means (standard deviations) for categorical variables. *p*-values were calculated using the Chi-square test or Fisher’s exact test for categorical variables and the *t*-test or Wilcoxon rank-sum test for continuous variables. IUP, intrauterine pregnancy; CS, cesarean section; BMI, body mass index; PPH, postpartum hemorrhage. ^1^ History of delivery: defined as term and preterm delivery. ^2^ IUP: defined as term, preterm delivery, and abortion.

**Table 2:** Comparison of pregnancy outcomes according to the location of the placenta.

Characteristic	Location of the Placenta		*p*-Value
	Anterior, *n* = 209	Posterior, *n* = 572	
Preop Hgb (mg/dL)	11.3 ± 1.5	11.4 ± 1.4	0.47
POD#1 Hgb (mg/dL)	10.1 ± 1.6	10.4 ± 1.6	0.056
POD#3 Hgb (mg/dL)	9.3 ± 1.4	9.5 ± 1.4	0.17
Transfusion (%)	120 (57.4%)	210 (36.7%)	<0.001
>3 units of packed RBCs (%)	38 (34.9%)	32 (17.8%)	0.001
>5 units of packed RBCs (%)	25 (22.9%)	17 (9.4%)	0.002
>10 units of packed RBCs (%)	11 (10.1%)	1 (0.6%)	<0.001
EBL (cc)	974.9 ± 1287.2	639.4 ± 450.4	<0.001
PAS ^1^ (%)	40 (19.1%)	50 (8.8%)	<0.001
Placental abruption (%)	6 (2.9%)	12 (2.1%)	0.59
PPH (%)	14 (14.7%)	31 (8.8%)	0.085
DIC (%)	3 (1.4%)	2 (0.3%)	0.122
ICU admission (%)	0 (0.0%)	2 (0.3%)	>0.999

Values are presented as numbers (percentages) and means (standard deviations) for the categorical variables. *p*-values were calculated using the Chi-square test or Fisher’s exact test for categorical variables. Preop, preoperative; Hgb, hemoglobin; RBC, red blood cell; POD#1, postoperative day 1; POD#3; postoperative day 3, EBL, estimated blood loss; PAS, placenta accreta syndrome; PPH, postpartum hemorrhage; DIC, disseminated intravascular coagulation; ICU, intensive care unit. ^1^ PAS is defined as placenta accreta, placenta increta, or placenta percreta.

**Table 3:** Neonatal outcomes.

Characteristic	Location of the Placenta		*p*-Value
	Anterior, *n* = 209	Posterior, *n* = 572	
Birth weight (g)	2689.3 ± 704.9	2815.7 ± 623.5	0.038
Neonatal sex (%)			0.795
Male	50 (52.6%)	181 (51.1%)	
Female	45 (47.4%)	173 (48.9%)	
Apgar score < 7 (1 min) (%)	58 (27.8%)	138 (24.1%)	0.301
Apgar score < 7 (5 min) (%)	21 (10.0%)	40 (7.0%)	0.159
Fetal presentation (%)			<0.001
Vertex	74 (79.6%)	327 (92.4%)	
Non-vertex (Breech, transverse position)	19 (20.4%)	27 (7.6%)	

Values are presented as numbers (percentages) and means (standard deviations) for the categorical variables. *p*-values were calculated using the Chi-square test or Fisher’s exact test for categorical variables.

**Table 4:** Obstetric adverse outcomes with anterior previa.

Variable	OR	95% CI	*p*-Value
Transfusion	2.23	1.50–3.33	<0.001
>3 units packed RBCs	1.67	0.85–3.39	0.14
>5 units packed RBCs	1.82	0.81–4.4	0.15
PAS	2.16	1.21–3.97	0.009
Admission for bleeding	1.3	0.88–1.94	0.19
Non-vertex fetal presentation	2.47	1.09–5.88	0.031

ORs are calculated using logistic regression. RBC, red blood cell; OR, odds ratio; CI, confidence interval; PAS, placenta accreta spectrum.

## Data Availability

All data generated or analyzed during this study are included in this article. Further enquiries can be directed to the corresponding author.
